# Plant Pathogenic Microorganisms: State-of-the-Art Research in Spain

**DOI:** 10.3390/microorganisms11030816

**Published:** 2023-03-22

**Authors:** Elvira Fiallo-Olivé, Ana Palacio-Bielsa, Soledad Sacristán

**Affiliations:** 1 Instituto de Hortofruticultura Subtropical y Mediterránea “La Mayora” (IHSM-UMA-CSIC), Consejo Superior de Investigaciones Científicas, 29750 Algarrobo-Costa, Spain; 2 Centro de Investigación y Tecnología Agroalimentaria de Aragón, Departamento de Sistemas Agrícolas, Forestales y Medio Ambiente, Instituto Agroalimentario de Aragón—IA2, CITA—Universidad de Zaragoza, 50059 Zaragoza, Spain; 3 Centro de Biotecnología y Genómica de Plantas, Instituto Nacional de Investigación y Tecnología Agraria y Alimentaria (INIA/CSIC), Universidad Politécnica de Madrid (UPM), Campus de Montegancedo UPM, 28223 Pozuelo de Alarcón, Spain; 4 Departamento de Biotecnología-Biología Vegetal, Escuela Técnica Superior de Ingeniería Agronómica, Alimentaría y de Biosistemas, Universidad Politécnica de Madrid (UPM), 28040 Madrid, Spain

Pathogenic microorganisms, including fungi, oomycetes, bacteria, viruses, and viroids, constitute a serious threat to agriculture worldwide. In Spain, one of the countries with the highest proportion of agricultural gross domestic product in Europe, the agri-food industry is the main manufacturing activity. Consequently, the presence and emergence of microorganisms causing serious plant diseases to economically important crops is especially relevant. In line with this, Spain has an important number of research groups interested in plant pathology, with scientists working on many aspects of pathogenic microorganism–plant interactions, from the basic aspects to more applied studies. In recent years, numerous important advancements have been achieved by scientists working in Spain in terms of the biological and molecular characterization of plant pathogenic microorganisms, in elucidating mechanisms of microbe pathogenesis, plant resistance to microbe infection, and plant–microbe–vector interactions. All these new achievements have provided essential knowledge for agricultural researchers worldwide.

The aim of this Special Issue was to provide a platform for Spanish researchers interested in plant pathogenic microorganisms to share their recent results related to microbe–plant host interactions, microbe–vector interactions, microbe–microbe interactions, evolution, ecology, and control strategies. A total of 11 papers have been contributed by 47 authors to the Issue, including 8 research articles and 3 reviews.

The availability of sensitive and accurate pathogen detection methods is crucial for plant pathologists and disease management. In this Special Issue, the paper by Quintana et al. [1] focused on this topic. The authors assessed different DNA extraction methods for the detection of the uncultured bacterium ‘*Candidatus* Liberibacter solanacearum’ (CaLsol) by qPCR in the psyllid vector. They also evaluated the influence on the detection of CaLsol by qPCR in *Bactericera trigonica* of four specimen preparations (entire body, ground, cut-off head, and punctured abdomen) and seven DNA extraction methods. Although optimum results were obtained through grinding, destructive procedures were not essential in order to detect CaLsol. The HotSHOT method was accurate, fast, simple, and sufficiently sensitive to detect the bacterium within the vector. This work provides a valuable guide when choosing a method to detect CaLsol in vectors according to the purpose of the study.

The knowledge of the presence and distribution of plant pathogens is a key aspect in the study of their epidemiology. In this Special Issue, three papers cover aspects related to the detection, molecular and biological characterization, and distribution of plant pathogens in Spain.

Fiallo-Olivé et al. [2] detected the mastrevirus sweet potato symptomless virus 1 in sweet potato plants for the first time in Spain (southern continental Spain and the Canary Islands) and Europe. Sequence analysis of the full-length genomes of isolates from Spain showed novel molecular features. Additionally, in this work, the first agroinfectious clone for the virus was developed, and infectivity assays showed that it was able to asymptomatically infect *Nicotiana benthamiana*, *Ipomoea nil*, *I. setosa*, and sweet potato.

In their contribution to this Special Issue, Fernández-Sanz et al. [3] studied the biochemical diversity and pathogenicity of the bacterium *Pseudomonas viridiflava* from common bean and weeds in Northern Spain and carried out a phylogenetic analysis. Regardless of their origin, the isolates displayed biochemical diversity. Phylogenetic analysis revealed two clusters of strains containing different pathogenicity islands, with no correlation observed for the plant host, biochemical profile, or pathogenicity. Ten new weed genera/species were identified as the new host of the bacterium, and more than half of the weed isolates were pathogenic in common bean. This work supports the role of weeds as reservoirs of *P. viridiflava* and a source of inoculum for common bean infection, further highlighting the role of weeds on the epidemiology of the disease.

The work by Hernández et al. [4] is focused on the fungal pathogens associated with aerial symptoms of avocado in Tenerife (Canary Islands). Avocado is one of the most important crops in this region, and the production area has been continuously increasing in recent years, with the rise of diseases associated with symptoms such as dieback, the external necrosis of branches and inflorescences, cankers on branches and trunks, or the stem-end rot of fruits. Hernández et al. obtained 297 isolates from 158 vegetal samples collected from 2018 to 2022 that were identified using morphological and molecular methods. Most of the isolates were the Botryosphaeriaceae species, and the authors have reported the first occurrence of *Lasiodiplodia brasiliensis* as an avocado dieback-producing pathogen and *N. cryptoaustrale*/*stellenboschiana* as a cause of dieback and stem-end rot symptoms in avocado.

The ability of plant pathogens to adapt to environmental conditions is of special concern since it has direct consequences on their survival and capacity to expand to new regions. The work by Álvarez et al. [5] is focused in *Ralstonia solanacearum*, a bacterial phytopathogen, originally from tropical and subtropical areas, whose ability to survive in temperate environments is of concern due to global warming. In this study, two strains from either cold or warm habitats were stressed by a simultaneous exposure to natural oligotrophy at low, temperate, or warm temperatures in environmental water. In their study, *R. solanacearum* adapted through different survival responses, irrespective of their cold or warm origin. In addition, starved, cold-induced viable but no culturable state, and/or resuscitated cells maintained virulence *in planta*. This work first describes the natural nutrient availability of environmental water favoring *R. solanacearum* survival, adaptations, and resuscitation in conditions that can be found in natural settings.

Understanding the mechanisms that pathogens use to sense and colonize the host has upmost importance to design control strategies that interfere with this process, as well as to evaluate the risk of pathogen adaptation to new hosts. Sena-Vélez et al. [6] have studied the chemotactic responses of bacteria from the genus *Xanthomonas* with different host ranges. The authors identified different chemotactic responses for carbon sources and apoplastic fluids depending on the *Xanthomonas* strain and the host plant from which the apoplastic fluid was derived. These differential chemotactic responses suggest that *Xanthomonas* strains sense host specific signals that facilitate their location and entry of stomatal openings or wounds.

Plant pathogens developed different strategies for their multiplication in the hosts and transmission between plants, ensuring their evolution. In this Special Issue, Martín-Hernández and Pagán [7] conducted comparative genomic approaches using the genus *Begomovirus* of plant viruses as a model. These analyses showed that terminal gene overlapping decreases the rate of virus evolution, which is associated with the lower frequency of both synonymous and nonsynonymous mutations. In contrast, terminal overlapping has little effect on the pace of virus evolution. The analyses carried out in this work support a role for gene overlapping in the evolution of begomoviruses and provide novel information on the factors that shape their genetic diversity.

Losses in crop yields due to disease need to be reduced in order to meet increasing global food demands associated with the growth of the human population. There is a well-recognized need to reduce the use of chemical pesticides and, therefore, new environmentally friendly control strategies to combat crop disease should be developed and implemented. Focused on this theme, four papers have been published in this Special Issue.

Cabrefiga et al. [8] described an integrated approach for the control of *Alternaria* spp., the causal agent of apple leaf blotch and fruit spot disease, which recently appearance in Spain, where it causes important losses. This disease is difficult to control and requires a lot of treatments during the season. In addition, treatments must be carried out until the end of the season with the implications on fruit residues. For these reasons, an environmentally friendly strategy should be implemented. The results obtained in the present work indicate that the reduction in primary inoculum production, through the removal of winter fallen leaves and also with the treatment of leaves with the biological agent *Trichoderma asperellum*, is an important key to increase the control efficiency and to help in the reduction in phytosanitary products.

Quetglas et al. [9] analyzed the control action plan implemented in the Balearic Islands after the detection, in October 2016, of *Xylella fastidiosa*, a quarantine bacterium in the European Union. The pathogen is already naturalized in the territory, so the application of eradication or containment measures are not substantiated in epidemiological and evolutionary terms. In this review, the authors describe which control measures may or may not work for the epidemiological situations of *X. fastidiosa* on each island and how the perception of control measures has been changing as knowledge of the epidemiological situation has increased.

Badosa et al. [10] have reviewed their twenty-year experience in the field of plant diseases control, in which they have improved the methods and technologies for the in vitro or *in planta* screening of a large number of linear and cyclic peptides as well as peptide conjugates. These screening methods include the assessment of antibacterial, hemolytic, and phytotoxicity activities, and also for their plant defense elicitor capacity. As a result, the authors have been able to identify sequences that can be considered promising candidates to develop effective phytosanitary plant protection products for their use in agriculture.

Bonaterra et al. [11] have reviewed the role of biological control as a sustainable alternative or complement to conventional plant protection products for the management of fungal and bacterial plant diseases. Some of the most intensively studied biological control agents are bacteria that use multiple mechanisms involved in limiting the development of plant disease. Although several bacteria-based plant protection products have already been registered and commercialized, efforts are still required to increase the number of products available on the market. This review shows some relevant examples of known bacterial biocontrol agents. The importance of the selection process and key steps in the development of such agents is highlighted, and some improvement approaches and future trends are considered.
